# Wiggle-match radiocarbon dating of the Taupo eruption

**DOI:** 10.1038/s41467-019-12532-8

**Published:** 2019-10-11

**Authors:** Alan G. Hogg, Colin J. N. Wilson, David J. Lowe, Chris S. M. Turney, Paul White, Andrew M. Lorrey, Sturt W. Manning, Jonathan G. Palmer, Sarah Bury, Julie Brown, John Southon, Fiona Petchey

**Affiliations:** 1 Radiocarbon Dating Laboratory, University of Waikato, Hamilton, New Zealand; 2 School of Geography, Environment and Earth Sciences, Victoria University, Wellington, New Zealand; 3 School of Science (Earth Sciences), University of Waikato, Hamilton, New Zealand; 4 Palaeontology, Geobiology and Earth Archives Research Centre, School of Biological, Earth and Environmental Sciences, University of New South Wales, Sydney, New South Wales Australia; 5 GNS Science, Wairakei, New Zealand; 6 National Institute of Water and Atmospheric Research, Auckland, New Zealand; 7 Cornell Tree Ring Laboratory, Department of Classics, Cornell University, Ithaca, New York 14853 USA; 8 National Institute of Water and Atmospheric Research, Wellington, New Zealand; 9 Department of Earth System Science, University of California, Irvine, California USA

**Keywords:** Volcanology, Geochemistry, Volcanology

**Arising from** Richard N. Holdaway et al. *Nature Communications* 10.1038/s41467-018-06357-0 (2018)

The Taupo eruption^1^ deposit is an isochronous marker bed that spans much of New Zealand’s North Island and pre-dates human arrival^2^. Holdaway et al.^3^ (HDK18 hereafter) propose that the current Taupo eruption date is inaccurate, and that the eruption occurred decades to two centuries after the published wiggle-match estimate of 232 ± 10 CE (2 SD)^4^ derived from a tanekaha (*Phyllocladus trichomanoides*) tree at the Pureora buried forest site^5,6^. HDK18 propose that trees growing at Pureora (and other near-source areas) that were killed and buried by the climactic ignimbrite event were affected by ^14^C-depleted (magmatic) CO_2_. HDK18’s proposal utilises a wide range of published ^14^C data, but their work results in assertions that are implausible. Four parts to their hypothesis are considered here.

The ^14^C-date compilation used by HDK18 to claim that the Pureora and other near-source dates are anomalously old is flawed. The dataset used to construct HDK18’s Fig. 1 is incomplete: at least 18 additional ages (including short-lived leaf and seed material)^7^ on Taupo eruptives from various sites (e.g., ref. ^8^) were not included. Most of the dates used in the figure have large errors and calibrated mean values extend between 650 CE and −100 CE, making them statistically indistinguishable and undermining the significance of any purported best fit correlation. This wide range of ages was a principal reason why wiggle-match dating of the Pureora buried forest logs was undertaken^4^. Ages in HDK18 (Supplementary Table S1), used to infer an age-vs.-distance relationship, represent a collation of data obtained over more than half-a-century from different laboratories, using differing dating methods (i.e., solid-carbon, gas proportional counting, liquid scintillation spectroscopy, accelerator mass spectrometry), differing pretreatment regimes (i.e., no pretreatment, acid–base–acid pretreatment, cellulose extraction), and differing age calculation procedures (i.e., non-Conventional Radiocarbon Age (CRA) vs. CRA). Indeed, many of the apparently anomalous oldest reported ages are from analyses dating to the 1950s–60s^9^. Even with modern techniques and consistent protocols, there remain inter-laboratory differences that preclude simple collation of ^14^C data sets. For example, Hogg et al.^4^ (Fig. 4) show that the Rafter and Waikato laboratory analyses, undertaken on wood derived from the same tanekaha tree-ring chronology^6^, have a systematic offset, with Rafter analyses, which dominate HDK18 (Supplementary Table S1), on average 40 years younger. Of critical importance, the Waikato study circumvented such laboratory bias by analysing a 250-year series of contiguous decadal ^14^C dates from the Pureora tanekaha tree and wiggle-matched them against known calendar-age kauri (*Agathis australis*) to derive a date for the eruption of 232 ± 10 CE^4^.

**Fig. 1:**
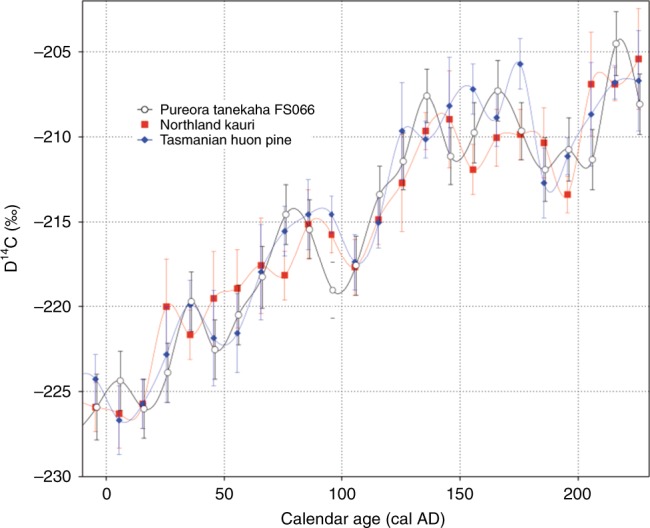
Comparisons of radiocarbon concentrations from New Zealand and Tasmania trees. Radiocarbon concentration (D^14^C, 1 *σ* error bars) plotted against calendar age for the Pureora tanekaha tree FS066^4^ together with Northland kauri^14^ and Tasmanian huon pine^15^. It should be noted that the vertical axis title in HDK18’s Fig. 3 is incorrect—it should read D^14^C, as above, not Δ^14^C, which is age-corrected ^14^C concentration

Relationships between the dates in HDK18’s Supplementary Table S1 (36 values), Supplementary Fig. S2 (45 values) and the Taupo eruption deposits are also unclear, with the stratigraphic context often lacking, impairing the value of the age estimates. An example of best practice is from a section^10^ at Kaipo bog, far removed from any possible magmatic ^14^C contamination^3^, which incorporates the Taupo eruption deposits. Here, stratigraphically ordered, independent age points (37 local ^14^C ages and 16 tephrochronological ages) were used^10^ to derive dates (not cited by HDK18) for the Taupo layer of 231 ± 12 CE (OxCal) and 251 ± 51 CE with a weighted-mean date of 240 CE (Bacon-software-derived), statistically identical to the Pureora wiggle-match estimate^4^.

The potential impact of injected ^14^C-depleted magmatic CO_2_ on reservoir ages in Lake Taupo (and the Waikato River draining the lake) is documented^11^. HDK18 present ^14^C dates of organic materials from this area, i.e., within 60 km of the Taupo eruption source (HDK18, Fig. 3), and propose that these dates are biased towards older ages by CO_2_ degassed from groundwater. We discount this proposition at the Pureora forest site for several reasons. First, deep, ^14^C-depleted groundwater is most unlikely to have affected the Pureora site, as it lies at 550 m above sea level^5^, in a separate catchment from that of the Waikato River, and is ~300 m above and 20 km distant from the Waikato River at its nearest point. Second, the site is ~200 m above the level of Lake Taupo and lies west of the watershed between it and the Taupo basin. Groundwater at the site is sourced from local rainfall (1.8 m of rainfall per year^5^). Third, the Pureora area also shows no traces of young faulting^12^ that could have channelled putative magmatic CO_2_. Fourth, the mechanism of gaseous exchange to introduce ^14^C-depleted carbon into groundwater at the Pureora site is most unlikely. Groundwater flow at the site will be dominated by vertically downwards flow of rainfall recharge from the soil layers to deeper units and thus atmospheric CO_2_ must dominate carbon dioxide flux at the site. The notion that magmatic carbon could be introduced into groundwater of the Pureora site from magmatic sources beneath Taupo volcano (or anywhere in the central North Island), or somehow be introduced (against gravity) from the Waikato River water, is implausible.

HDK18 state that in the Pureora tanekaha tree-ring record, ^14^C levels plateaued or declined as the eruption approached (p. 5, Fig. 3 caption), and that after ~125 years (Fig. 3a), linear relationships with the actual tree age broke down: the tree continued to grow but ^14^C ages of the newly accreted wood were static (p. 4). However, the fitting of straight-line functions to ^14^C concentrations is meaningless, as non-linearity in ^14^C levels is universally recognised and underpins international calibration curves (e.g., SHCal13^13^) and wiggle matching for age correlations^4^. Here we re-plot the Pureora tanekaha ^14^C data against known calendar-age data from Northland (northernmost North Island) kauri^14^ and Tasmanian huon pine (*Lagarostrobos franklinii*)^13,15^ (Fig. 1). Although there is a general decline in ^14^C levels towards the time of death of the Pureora tanekaha tree (spanning ~50 years; Fig. 1), the contemporaneous kauri and huon pine ^14^C levels similarly decline, independent of any Taupo-proximal magmatic CO_2_ emissions. What HDK18 assert as evidence for isotopic dilution is simply a ^14^C wiggle in atmospheric ^14^C common to all three data sets.

In addition, HDK18 (Fig. 3a) propose a trend of lowered ^14^C levels for ~125 years before the Taupo eruption. If correct, one would expect wiggle matching to derive a younger date for the eruption if the ^14^C data from this 125-year interval were excluded from the wiggle matching. We thus divided the Pureora tanekaha dates into two sets (Table 1): an inner fraction, i.e., dates in the range 125.5–245.5 years before the eruption that HDK18 consider is linear with tree age, and an outer fraction, i.e., dates in the range 5.5–115.5 years before the eruption that HDK18 claim to be nonlinear as a result of ^14^C dilution. The two sets were then wiggle-matched against SHCal13^13^. The two sets considered separately give statistically identical model eruption dates both to each other and to the full 250-year dataset.

**Table 1:** Impact on the Taupo eruption date estimate

Wiggle match (utilising SHCal13^13^ calibration curve)	No. of analyses	Wk centre ring (years before eruption)^a^	Calendar age range (Mean cal. age) (CE, 95.4% prob.)	Am^b^ (%)	A < 60^c^ (Outliers^d^) (%)
Wk Pureora tanekaha ^14^C ages >125 years before last extant tree ring and eruption	12	125.5–245.5	220–240 (230 ± 10)	89.9	4 (4)
Wk Pureora tanekaha ^14^C ages <125 years before last extant tree ring and eruption	13	5.5–115.5	224–241 (233 ± 8)	100.0	4 (4)
All Wk Pureora tanekaha ^14^C ages	25	5.5–245.5	226–238 (232 ± 6)	98.7	8 (8)

Impact on the Taupo eruption date estimate as a result of dividing the 250-year Wk Pureora tanekaha ^14^C data series into two sets: an inner fraction, i.e., dates in the range 125.5–245.5 years before the eruption that HDK18 consider is linear with tree age, and an outer fraction, i.e., dates in the range 5.5–115.5 years before the eruption that HDK18 claim to be nonlinear as a result of ^14^C dilution

^a^Ring numbers from Hogg et al.^4^ (Table 1 in their study)

^b^Model agreement index. The agreement for the model as a whole. Ideally, the value should be ~100% and should be >60% (a threshold value close to the 5% confidence levels in a *χ*^2^-test). No reservoir offset function (Delta_R) applied

^c^Percentage of individual dates where the agreement index is below 60%

^d^Percentage outliers, where an outlier, detected by ‘outlier analysis’, has a posterior probability of >0.05 (prior probability of a date being an outlier set at 0.05)

HDK18’s analysis of the Pureora tanekaha tree δ^13^C record is flawed for two reasons. First, the Pureora tanekaha did not have at least 50 inner rings sampled, hence the lack of the so-called juvenile effect (increasing δ^13^C values as a juvenile: e.g., Supplementary Fig. 1), which will have influenced the shape of the δ^13^C record. Second, the Pureora tanekaha δ^13^C data, stated as anomalously high by HDK18, were obtained from the α-cellulose wood fraction with the CO_2_ produced by a through-flow combustion system, which together displace mean δ^13^C data to less negative values over those from the whole-wood fraction used by HDK18 by ~2‰ (Supplementary Table 1 and Supplementary Note 1). HDK18’s further statement that the Pureora tanekaha δ^13^C measurements are significantly higher than those of New Zealand forest trees (p. 4) is also not correct. For example, the outermost Pureora tanekaha rings yield cellulose δ^13^C values ~2‰ lower than the outermost rings from a kauri tree (e.g., Supplementary Fig. 1). The Pureora tanekaha δ^13^C values are neither anomalously high nor do they reflect any magmatic carbon input.

In conclusion, HDK18’s proposal^3^ that the Taupo eruption is decades to centuries younger than 232 ± 10 CE is unsound. Although ^14^C-depleted materials are associated with magmatic degassing^11^, the context and consistency of any radiocarbon dates indicate whether a robust and accurate age estimate has been attained. The 250-year ^14^C wiggle-match against SHCal13 presented here reinforces the view that 232 ± 10 CE^4^ remains the most accurate and precise age estimate for the Taupo eruption, and we conclude there is no evidence for anomalously older ages near the Taupo volcano. We re-assert that radiocarbon wiggle matching to refine volcanic event chronologies, especially where sequential ^14^C dates and Bayesian modelling form the basis of the event timing, remains an accurate and invaluable dating tool.

## Supplementary information

Supplementary Information

## Data Availability

All data generated for this study are included in Supplementary Table 1. All other data plotted are from the relevant published and cited papers.
