# Supplementary material for: Wiggle-match radiocarbon dating of the Taupo eruption
Source: Nat Commun. 2019 Oct 11;10:4669. doi: 10.1038/s41467-019-12532-8 (PMC6788995; doi:10.1038/s41467-019-12532-8)
Supplement: Supplementary file 1 — Supplementary Information [file 41467_2019_12532_MOESM1_ESM.pdf]

Supplementary Information for:

Wiggle-match radiocarbon dating of the Taupo eruption

by Hogg et al.

Supplementary figure

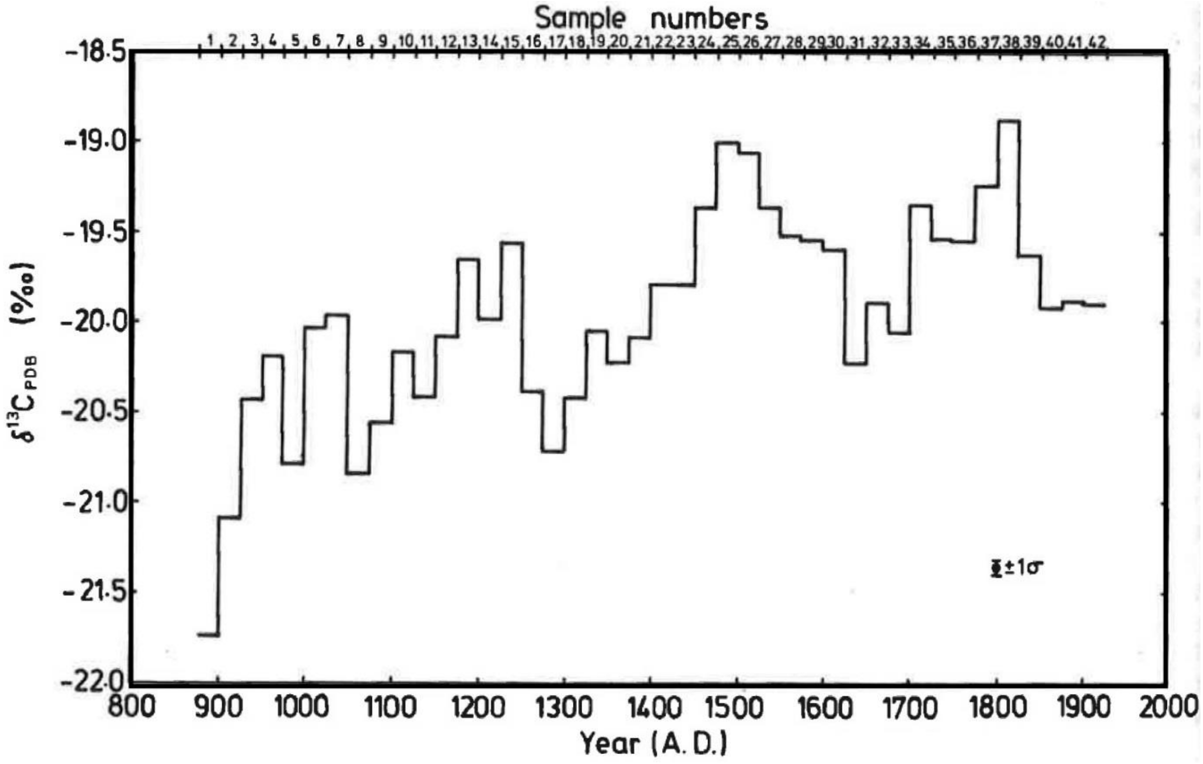

Supplementary Figure 1. Kauri tree-ring $\delta^{13}C$ levels plotted against year of growth (from Grinsted and Wilson (ref. 1, their Figure 2, p. 56) to show that the Pureora tanekaha $\delta^{13}C$ values are not significantly higher than those of New Zealand forest trees as stated by HDK18[2]. Kauri cellulose outer ring $\delta^{13}C$ values reach as high as -18.9‰, which is markedly higher than Pureora tanekaha outer ring $\delta^{13}C$, with a high value of -20.0‰. Note the juvenile effect where $\delta^{13}C$ values initially increase rapidly[3].

Supplementary table

Supplementary Table 1. δ^{13}C data derived from two wood fractions prepared from tree-ring samples from the Pureora buried forest tanekaha tree FS066[4] in comparison with matai and rimu. The measurements were measured in duplicate on solid samples by NIWA, Wellington, using a Thermo Fisher Scientific V Plus continuous flow isotope ratio mass spectrometer linked to a Flash 2000 elemental analyser using a MAS 200 R autosampler.

**Sample (geographic location)***	**Lab no. (Wk)**	**Wk δ^{13}C data from Hogg et al. (2012)[4] (‰)**	**Whole Wood fraction δ^{13}C (‰)**	**Alpha-cellulose fraction δ^{13}C (‰)**	**Whole wood minus alpha-cellulose (‰)**	**NIWA minus Wk alpha-cellulose fraction δ^{13}C (‰)**
Tanekaha rings 1491-1500 (Pureora, 5.5 yrs)	23140	-20.5	-22.29 ± 0.37	-21.05 ± 0.02	-1.23	0.55
Tanekaha rings 1461-1470 (Pureora, 35.5 yrs)	23143	-20.6	-22.63 ± 0.49	-20.99 ± 0.04	-1.64	0.39
Tanekaha rings 1251-1260 (Pureora, 245.5 yrs)	22980	-22.0	-23.7 ± 0.03	-22.51 ± 0.25	-1.19	0.51
Matai¶ (near Haast, Westland)	48309	n.a.	-23.88± 0.06	-22.46 ± 0.06	-1.42	
Rimu¶ (near Haast, Westland)	48310	n.a.	-23.71± 0.15	-21.93 ± 0.01	-1.78	

* For Pureora samples, average years before the eruption
¶ Ring numbers unknown. Matai and rimu samples supplied by A. Davies, Tréology Limited

Supplementary notes

Supplementary Note 1

The Pureora tanekaha δ^{13}C data from Hogg et al.[4] were obtained as part of the ^{14}C-dating process to correct isotopic fractionation in δ^{14}C, and should not have been used for any other purpose. The measurements were made on the alpha-cellulose wood fraction using a Europa Scientific Penta 20/20 Isotope Ratio Mass Spectrometer (IRMS) from CO_2 gas prepared via a through-flow combustion system, which does not necessarily produce δ^{13}C data that are directly comparable with other analytical methods. The HDK18 tree-ring material was submitted to Iso-Trace Ltd, Dunedin, as powdered untreated wood, and the analyses were performed on an Elemental Analyser (EA) with Isoprime or 20/20 IRMS (EA-IRMS, R. Van Hale pers. comm., 2018), which does not use the same type of through-flow combustion system to produce the CO_2.

To show that the Pureora tanekaha alpha-cellulose $\delta^{13}C$ values are not anomalously high because of proposed isotopic dilution, as proposed by HDK18[2], we re-measured $\delta^{13}C$ in three of the Pureora tanekaha tree (FS066) decadal samples used in the Hogg et al.[4] study and compared these with measurements from matai and rimu growing near Haast, Westland (Supplementary Table 1). For the Pureora tanekaha we chose two of the outer, younger, decades and, for comparison, an inner, older sample (details in Supplementary Table 1).

The results for the Pureora whole wood fractions range from -22.3 to -23.7‰, with matai and rimu slightly lower, ranging from -23.7 to -23.9‰ and similar to values for the Pureora tanekaha inner samples (-23.7‰). It should be noted that the ring numbers for the matai and rimu samples are unknown – they could be derived from either inner or outer rings. The values for the alpha-cellulose fractions for all five samples are markedly higher than those for untreated wood for both Pureora tanekaha (average of 1.35‰), matai (1.42‰), and rimu (1.78‰). The difference between the NIWA and Waikato Pureora tanekaha measurements (average of 0.48‰) reflects the two different methods for preparing the CO_2 for measurement (i.e. the use of an elemental analyser at NIWA compared with a through-flow combustion system at Waikato).

The whole wood $\delta^{13}C$ measurements for the younger outer Pureora tanekaha rings (average of -22.5‰), stated by HDK18[2] to be anomalously high because of isotopic dilution, are actually very similar to the values of HDK18's Glenmore Matai Tree 2 outer rings (-22.7‰, HDK18 supplementary table 3). The measurements in Supplementary Table 1 show conclusively that HDK18's statement that the Pureora tanekaha measurements are anomalously high throughout is inaccurate, with the Pureora tanekaha data being higher, not because of isotopic dilution but because HDK18 not only analysed different species (matai and rimu versus tanekaha), but also analysed different wood fractions (whole wood versus alpha-cellulose) and used different methods of analysis (EA-IRMS versus a through-flow combustion system).

Supplementary references

1. Grinstead, M. J. & Wilson, A. T. Variations of $^{13}C/^{12}C$ ratio in cellulose of *Agathis australis* (kauri) and climatic change in New Zealand during the last millennium. *N.Z. J. Sci.* **22**, 55-61 (1979).
2. Holdaway, R. N., Duffy, B. & Kennedy, B. Evidence for magmatic carbon bias in ^{14}C dating of the Taupo and other major eruptions. *Nat. Commun.* **9**, 4110 (2018).

3. Gagen, M., McCarroll, D., Robertson, I., Loader, N. J. & Jalkanen, R. Do tree ring $\delta^{13}C$ series from *Pinus sylvestris* in northern Fennoscandia contain long-term non-climatic trends? *Chem. Geol.* **252**, 42-51 (2008).
4. Hogg, A. G., Lowe, D. J., Palmer, J. G., Boswijk, G. & Bronk Ramsey, C. J. Revised calendar date for the Taupo eruption derived by ^{14}C wiggle-matching using a New Zealand kauri ^{14}C calibration data set. *The Holocene* **22**, 439-449 (2012).
